# On the Treatment of Dysentery

**Published:** 1853

**Authors:** 

**Affiliations:** Surgeon 37th Regiment


*On the Treatment of Dysentery.*—By Dr. Cameron, Surgeon 37th Regiment.

Dysentery is generally seen as colonitis in Ceylon, and very rarely attended with that hepatic complication so much talked of elsewhere. The treatment pursued has been local depletion by leeches to the abdomen and anus, the internal use of Dover's powder and ipecacuanha, in doses of three grains each, every third hour, during the early stage, and afterwards combinations of opium with kino, chalk, tannan, acetate of lead, &c., according to circumstances,

In all severe cases, I have for many years past conjoined quinine in moderate doses with the other remedies, giving to the extent of twelve or sixteen grains in the twenty-four hours, and, I am persuaded, with the best results as regards the patient's strength, and the prevention of sloughing of the mucous membrane. I attach much importance, also, to the support of the patient from the commencement. Sick men often die of starvation as much as anything else. I generally give a cup of arrow-root twice a-day for the first three days, and after that, continue it, with chicken broth, for dinner and drink. Opiate injections are commonly used. I have occasionally derived benefit from strong solutions of nitrate of silver thrown into the colon, but with the ordinary apparatus it is nearly impossible to use the remedy effectively, owing to decomposition.

*Med. Times and Gazette*, Oct. 8, 1853, p. 366.

